# Performance of Solid-state Hybrid Energy-storage Device using Reduced Graphene-oxide Anchored Sol-gel Derived Ni/NiO Nanocomposite

**DOI:** 10.1038/s41598-017-15444-z

**Published:** 2017-11-10

**Authors:** Himadri Tanaya Das, Kamaraj Mahendraprabhu, Thandavarayan Maiyalagan, Perumal Elumalai

**Affiliations:** 1 Electrochemical Energy and Sensors Lab, Department of Green Energy Technology, Madanjeet School of Green Energy Technologies, Pondicherry University, Puducherry, 605014 India; 2 SRM Research Institute & Department of Chemistry, SRM University, Kattankulathur, Chennai 603203 India; 3 Present Address: Department of Bioelectronics and Biosensors, Alagappa University, Karaikudi, 630003 Tamilnadu India

## Abstract

The influence of (nickel nitrate/citric acid) mole ratio on the formation of sol-gel end products was examined. The formed Ni/NiO nanoparticle was anchored on to reduced graphene-oxide (rGO) by means of probe sonication. It was found that the sample obtained from the (1:1) nickel ion: citric acid (Ni^2+^: CA) mole ratio resulted in a high specific capacity of 158 C/g among all (Ni^2+^: CA) ratios examined. By anchoring Ni/NiO on to the rGO resulted in enhanced specific capacity of as high as 335 C/g along with improved cycling stability, high rate capability and Coulombic efficiency. The high conductivity and increased surface area seemed responsible for enhanced electrochemical performances of the Ni/NiO@rGO nanocomposite. A solid-state hybrid energy-storage device consisting of the Ni/NiO@rGO (NR_2_) as a positive electrode and the rGO as negative electrode exhibited enhanced energy and power densities. Lighting of LED was demonstrated by using three proto-type (NR_2_^(+)^|| rGO^(−)^) hybrid devices connected in series.

## Introduction

The steep raise in global energy demand drives utilization of alternative and renewable energies. To meet the current energy demands and counter balance the depletion of the fossil fuels, the energy production and storage should go hand-in-hand. In this regard, the electrochemical energy storage devices such as batteries and supercapacitors are considered as a reliable technology. Among these two, the supercapacitors seem attractive energy-storage device as it can find its usage in peak power demands, backup power sources for computer memory, hybrid vehicles etc. The other attractive features of the supercapacitors are flexible operating temperature, high durability, low cost and portability. In fact, the supercapacitors can bridge the power gap between the batteries and the conventional capacitors^1^.

Based on the principle of charge storage, the supercapacitors are classified into two types i.e., electrical double layer capacitors (EDLCs) and pseudocapacitors. While the electrostatic charge separation (non-Faradaic) is the basis for charge storage in the EDLCs, a definite Faradaic (redox) reaction is responsible for charge storage in the pseudocapacitors. Though the EDLCs have long-term stability, the pseudocapacitors are more profitable than EDLCs because of high specific capacitance. The EDLC property is mostly shown by carbon-derived materials such as carbon nanotube, carbon nanowires, graphene oxide (GO), reduced graphene-oxide (rGO) etc.^2–4^, while the pseudocapacitance is usually shown by metal oxides, hydroxides, and some redox polymers^5,6^. The ruthenium oxide (RuO_2_) has been exploited as an electrode material for pseudocapacitor, besides other metal oxides such as MoO_3_, V_2_O_5_, Bi_2_WO_6_^7–11^. However, due to the high cost of Ru, there is an urgent need for developing cheaper materials. Among the various transition metal-oxides such as MnO, FeO, CoO and NiO, the NiO has been considered as unique because of its attractive features such as good redox property, high theoretical capacity (1295 C g^−1^), relatively large abundant and hence cost less, environmental friendly, ease of synthesis and its property could be tuned by means of doping with other moieties and good stability in basic medium^12,13^. In addition, the performance of the NiO can be tuned by changing its morphology. Thus, NiO has been generated and applied for various applications. For example, Wu *et al*., have reported the sol-gel synthesis of nanostructured NiO by varying temperature, calcination time, pH etc.^14^. Zhang *et al*., reported that the specific capacitance of the porous NiO nanocolumns was significantly higher than that of the nanoslices or nanoplates^15^. Vijayakumar *et al*., have examined supercapacitor properties of NiO nanoflakes^16^. The mesoporous NiO nanourchins prepared by hydrothermal method resulted in better electrochemical activity than the NiO nanoflakes^17^. Quite recently, the Ni/NiO composites were also examined as dye-absorption material as well as anode material for Li-ion battery^18^. Liu *et al*., has reported ultrathin and lightweight 3D free-standing Ni@NiO membrane electrode for supercapacitor application^19^. However, the influence of amount of Ni in Ni/NiO nanocomposite on the electrochemical properties has not been examined for hybrid energy-storage device.

Currently, to improve the mechanical strength and provide conducting network to the base (metal oxides or hydroxides) material, conducting materials such as carbon nanotubes or reduced graphene-oxide (rGO) are embedded^20–23^. The anchoring of the base material on the exfoliated conducting matrix reduces the diffusion length of the material, leading to improved performance. Thus, several synthetic strategies are adopted to generate the rGO-based nanocomposites^23–26^. The rGO usually shows EDLC property. When the rGO is added to a pseudocapacitor material (ex. NiO), the resultant composite is expected to show better electrochemical performance. Therfore, in the present work, Ni/NiO nanocomposites are initially generated by a simple modified sol-gel (Pichini) process by varying nickel nitrate and citric acid (Ni^2+^:CA) mole ratio. The influence of Ni in the resultant Ni/NiO nanocomposites on electrochemical activity has been examined. Further, Ni/NiO@rGO nanocomposites were prepared and their hybrid energy-storage characteristics have been examined in detail.

## Results

### Crystal Structure and Morphology

**Figure 1:**
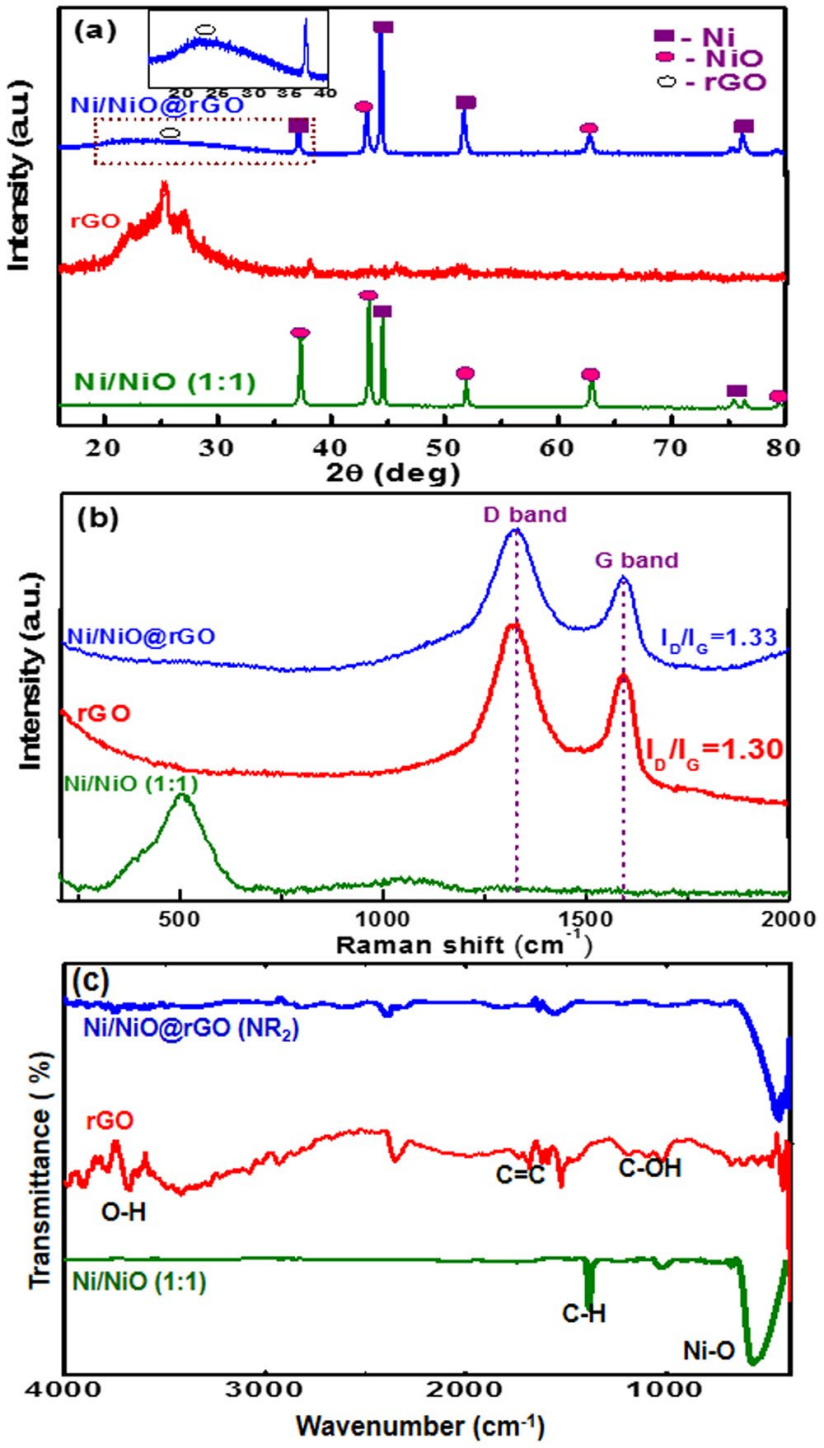
(**a**) XRD patterns, (**b**) Raman spectra and (**c**) FT-IR spectra of the Ni/NiO (1:1), rGO and NR_2 _samples.

Figure 1(a) shows the XRD patterns recorded for the Ni/NiO (1:1), rGO and Ni/NiO@rGO nanocomposites. The XRD pattern of the Ni/NiO sample obtained from the (1:1) (Ni^2+^:CA) mole ratio has the set of Bragg peaks located at diffraction angles (2*θ*), 44.5, 52.0 and 76 which are indexed to the cubic phase of Ni as per the standard JCPDS PDF # 89–7128^4^. The other set of Bragg peaks appeared at 37.4, 43.3, 62.9, 75.62 and 79.5° could be assigned to cubic phase of NiO as per the standard XRD pattern JCPDS # 73–1519^18,25^. The inset of Fig. 1 shows the enlarged XRD pattern in the 2*θ* range of 15–35°. The appearance of a broad peak centered at 2*θ* = 24.5° corresponds to (002) Bragg peak of carbon (JCPDS # 54–0501). The presence of such a broad peak implies the poor ordering of the graphene sheets along the stacking direction confirming that the rGO is comprised of a few layers of graphene^27^. Undoubtedly, the XRD pattern of the Ni/NiO@rGO (NR_2_) sample consists Bragg peaks of both the Ni/NiO and the rGO confirming composite nature. To substantiate the XRD data, Raman spectra of the samples were recorded and the obtained spectra are shown in Fig. 1(b). The Raman spectrum of the Ni/NiO (1:1) sample consists of single Raman shift at about 500 cm^−1^ which corresponds to the Ni-O bond^17^. The rGO sample has two Raman shifts at 1320 and 1593 cm^−1^ which correspond to the D and G bands of the rGO, respectively^27^. While the appearance of the D band is originated from the edges of the defected carbon layers due to breakdown of translational symmetry, the G band is attributed to the second-order scattering of the graphitic carbon. It is well known that the disorder in the rGO is measured by the intensity ratio of the D and the G bands. In the present case, the (I_D_/I_G_) is found to be 1.3. Such a high (I_D_/I_G_) value confirms that extensive oxidation and reduction have resulted in the GO, leading to large disorders in the rGO. Expectedly, the Raman spectrum of the Ni/NiO@rGO (NR_2_) nanocomposite consists of Raman shifts at 1322 and 1589 which corresponds to the D and the G bands of the rGO, respectively as discussed above. It is noted that the (I_D_/I_G_) ratio for the composite is 1.33, conforming the presence of large amount of reduction in the rGO. The NR_2_sample also shows a small hump at 500 cm^−1^ which corresponds to the Ni-O bond^17,21^. It is noted that the intensity of Raman shifts recorded for the NR_2_ is dominated with the high intense rGO peaks which submerged the NiO peak. The Raman spectra recorded for the NR_1_ and NR_3_ samples are given in supplementary material (Fig. S1a).

Figure 1(c) shows the FT-IR spectra recorded for the Ni/NiO, rGO and Ni/NiO@rGO nanocomposite (NR_2_). The strong peak appeared at 570 cm^−1^ confirms the presence of the Ni–O bond in the Ni/NiO and NR_2_ samples. The peak at 1000 cm^−1^ is characteristic for C–H bond which could have come from the precursor molecules or partially decomposed products of the precursor. The peak at around 1300 cm^−1^ is due to the presence of carbonyl band of a metal-carboxylate stretch (carboxyl group of citric acid coordinated to Ni). The FT-IR spectrum of the rGO shows peaks at 3422, 1528, 1147 cm^−1^ which are ascribed to the presence of O-H, C = C, C-OH bonds, respectively. The NR_2_ sample has the peaks at 1566 and 1128 cm^−1^ which are due to C = C and C-OH bonds of the rGO, and a sharp peak in the range of 450–500 cm^−1^ corresponding to the Ni-O bond. The IR spectra recorded for the NR_1_ and NR_3_ samples are given in supplementary material (Fig. S1b).

**Figure 2:**
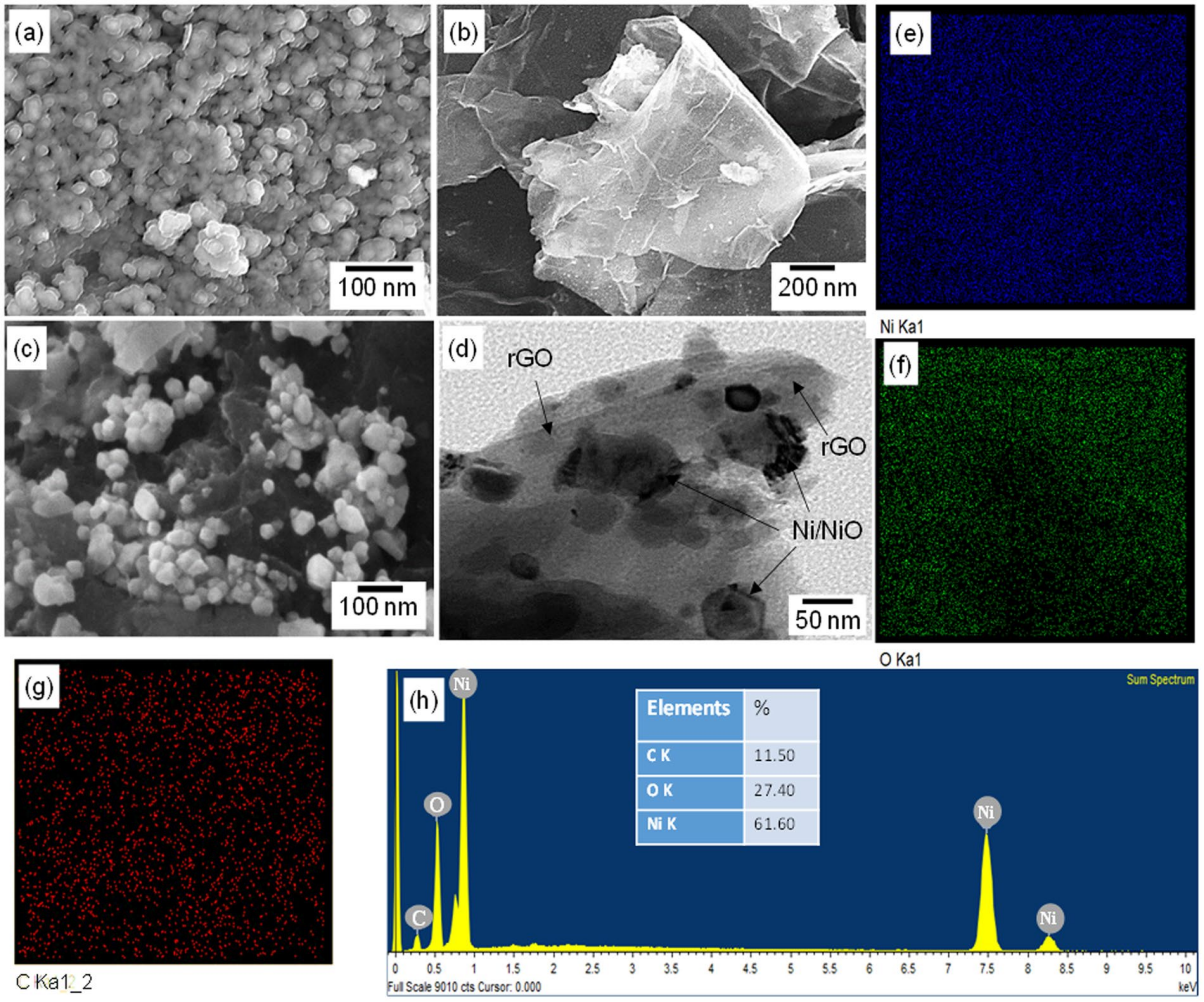
SEM images of (**a**) Ni/NiO (1:1), (**b**) rGO, (**c**) Composite NR_2_ and (**d**) TEM image of NR_2_. Elemental mappings (**e**–**f**) and EDAX profile of NR_2_.

Figure 2 shows the SEM images taken on the surface of the Ni/NiO (1:1), rGO and the representative Ni/NiO@rGO sample (NR_2_). It can be seen in Fig. 2(a) that the surface of the sample obtained from the (1:1) (Ni^2+^:CA) mole ratio consisted of almost uniform spherical grains with an average size of about 30 nm. The morphology of the rGO seems like folded curtain, forming overlapped compact structure rather than agglomerated sheets. The thickness of sheets was estimated to be less than 30 nm with a large dimension of over 2 μm. The folded structure with the large dimension of sheets is the typical morphology observed for the rGO^28^. Interestingly, the morphology of the Ni/NiO@rGO (NR_2_) composite consists of spherical Ni/NiO grains spread over the rGO nanosheets. It is clearly visible that the Ni/NiO grains are anchored on to the surface of the rGO nanosheets which has been substantiated by TEM image given in the Fig. 2(d). The TEM image also shows the homogenous distribution of the Ni/NiO grains onto the rGO sheets. The grain size obtained from TEM image nearly matches with that of the grain size obtained for the Ni/NiO from SEM. Further, to examine the distribution of elements, Ni, O, and C in the samples, the elemental mappings were done. Figure 2(e, f, g) show the obtained elemental mappings for Ni, O and C, respectively. It is seen that each element is homogenously distributed. The low intensity observed for the C is due to its limited amount in the nanocomposite (~11%). It should be noted that a simple probe sonication has resulted in such a homogenous distribution of Ni/NiO grains on the rGO nanosheets. It is bleived that the probe sonication has exfoliated the rGO layers sufficiently, then the Ni/NiO grains are anchored firmly on the rGO nanonetwork. The process of drying finally lead to formation of Ni/NiO@rGO nanocomposite. To substantiate the elemental mappings, the EDAX profile of the Ni/NiO@rGO (NR_2_) sample was recorded. The obtained EDAX profile is presented in Fig. 2(h). The presence of elements Ni, O and C is clearly seen. From the intensity of the peaks, the estimated amounts of Ni, O and C are found to be 62, 27 and 11%, respectively. Undoubtedly, the higher amount of Ni is due to the fact that the sample obtained from the (1:1) mole ratio had Ni/NiO in it. By the way, it was observed that the addition of the rGO to the Ni/NiO resulted a large reduction in saturation magnetization from 28 emu/g to 6 emu/g. The detailed hysteresis curves and its description have been given in the supplementary (Fig. S2). Thus, confirming the presence of rGO in the NR_2_ sample.

### Electrochemical Activity of Ni/NiO and Ni/NiO@rGO Nanocomposites

**Figure 3:**
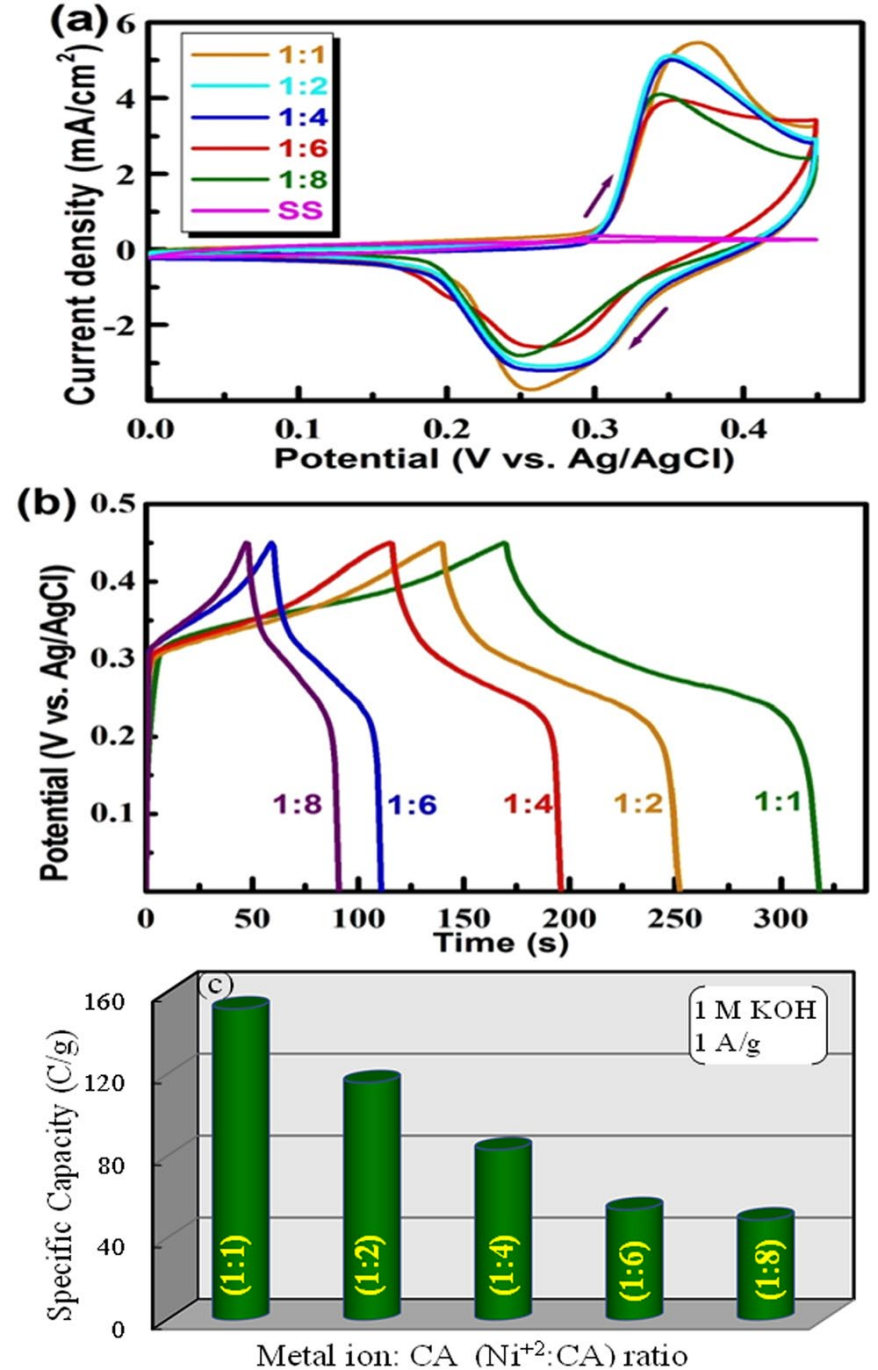
(**a**) Cyclic voltammograms at 5 mV/s, (**b**) Charge-discharge profiles at 1 A g^−1^, and (**c**) specific capacity recorded on the samples obtained from each of (Ni^2+^: CA) mole ratios in 1 M KOH.

The electrochemical activity of each sample was examined by cyclic voltammetry studies in 1 M KOH in three electrode configuration in the potential range of 0–0.45 V at a scan rate of 5 mV/s. The CV curves of all the samples obtained from each of (Ni^2+^: CA) mole ratio i.e., (1:1, 1:2, 1:4, 1:6 and 1:8) are shown in Fig. 3(a). It can be seen that a pair of redox peaks appears in all cases. The appearance of non-rectangular shape CV curves with strong redox peaks indicates that the Ni/NiO nanocomposites are electrochemically active and has undergone definite Faradaic reaction. The following Faradaic reactions are believed to proceed in KOH^28^:

1 $${\rm{NiO}}+{{\rm{OH}}}^{-}\rightleftarrows {\rm{NiOOH}}+{{\rm{e}}}^{-}$$

2 $${\rm{N}}{\rm{i}}({\rm{O}}{\rm{H}}{)}_{2}+{{\rm{O}}{\rm{H}}}^{-}\rightleftarrows {\rm{N}}{\rm{i}}{\rm{O}}{\rm{O}}{\rm{H}}+{{\rm{e}}}^{-}+{{\rm{H}}}_{2}{\rm{O}}$$

The forward peak appeared at 0.33 V is attributed to the formation of NiOOH by oxidation of NiO, while the reverse peak observed at 0.27 V is due to the reduction of NiOOH to Ni(OH)_2_. It is noted that the peak potential separation is 60 mV which is close to the value that, usually observed for an ideal reversible reaction 59 mV. Excitedly, the peak potential separation observed for the lower mole ratio samples is slightly higher, indicating a quasi-reversible nature. Indeed, it is noted that the sample obtained from the (1:1) mole ratio shows the highest peak current compared to the samples obtained from other (Ni^2+^:CA) mole ratios. The high current observed for the (1:1) Ni/NiO sample could be due to the presence of more amounts of nickel (Ni) metal which would have led to enhanced conductivity and facilitated efficient redox reaction. In addition, Ni metal would have also actively participated in the redox reaction as shown below^29^:

3 $${\rm{Ni}}+{{\rm{2OH}}}^{-}\to {\rm{Ni}}{({\rm{OH}})}_{2}+{{\rm{2e}}}^{-}$$

The appearance of a shoulder cathodic peak at 0.3 V confirms additional reaction occurring on the Ni/NiO (1:1) electrode. It is seen that the sample obtained from the (1:2) mole ratio electrode shows lower peak current than that of the sample obtained from the (1:1) mole ratio electrode, may be due to less amount of Ni metal in the sample. Wherein other samples, only NiO is present and responsible for the redox behavior, leading to very low peak current. It is noted that the SS substrate did not produce any significant current in KOH. Hence, the appearance of redox peaks is originated from the Ni/NiO samples. The high peak current observed for the Ni/NiO sample obtained from the (1:1) mole ratio implies that it can store more amount of charge.

Figure 3(b) shows the galvanostatic charge-discharge profiles for the samples obtained from each of the (1:1), (1:2), (1:4), (1:6), and (1:8) mole ratios recorded at a current density of 1 A/g. The uniformity of all charge-discharge profiles confirms that the redox reactions are highly reversible. The charge-discharge profiles have a notable plateau, thus conforming a battery-type behavior. A notable large charge/discharge times were exhibited by the Ni/NiO samples obtained from the (1:1) and (1:2) mole ratios, substantiating the above CV data. In addition, the charging and discharging times are not equal. It seems that during charging both Ni and NiO are converted to NiOOH, while discharging, the formed NiOOH is converting back to NiO or Ni(OH)_2_. Thus, disproportionate times are observed. During discharging process, it seems that some amount of NiOOH did not reduce to NiO or Ni(OH)_2_ which led to lower discharge time. In other samples, the charging and discharging times are nearly equal, confirming that only involvement of NiO in redox process. The specific capacity of all the electrodes was assessed from the charge-discharge profiles using the following formula^30^:

4 $$\,{C}_{s}=\frac{I\times t}{m}\,C/g$$

where *C*_*S*_ is specific capacity (C/g), *I* is discharging current (A), *∆t* is discharge time (s) and *m* is active mass (g). Figure 3(c) shows the dependence of specific capacity of Ni/NiO nanocomposites on the (Ni^2+^: CA) mole ratio. The (Ni^2+^: CA) mole ratio has a strong influence on the specific capacity of the resulted Ni/NiO samples. At higher (Ni^2+^: CA) mole ratios, lower the specific capacity has resulted and at lower (Ni^2+^: CA) mole ratio, higher the specific capacity has been observed. It can be seen that the Ni/NiO electrode obtained from the (1:1) mole ratio exhibits the highest specific capacity of 158 C/g, whereas the Ni/NiO electrodes obtained from the (1:2), (1:4), (1:6) and (1:8) mole ratios show in the range of 112–45 C/g. The high specific capacity of the Ni/NiO (1:1) electrode obtained from (1:1) mole ratio is believed to be involvement of both Ni and NiO in the Faradaic process. All other samples had either the lower amount of Ni or no Ni. Thus, only NiO is involved in the Faradaic process, obviously resulted in less specific capacity. It is noteworthy that tunable specific capacity can be obtained for Ni/NiO electrode by varying the (Ni^2+^: CA) mole ratio in the sol-gel preparation bath. Since the Ni/NiO electrode obtained from the (1:1) mole ratio exhibited the highest specific capacity, its further redox characteristics were examined in detail.

**Figure 4:**
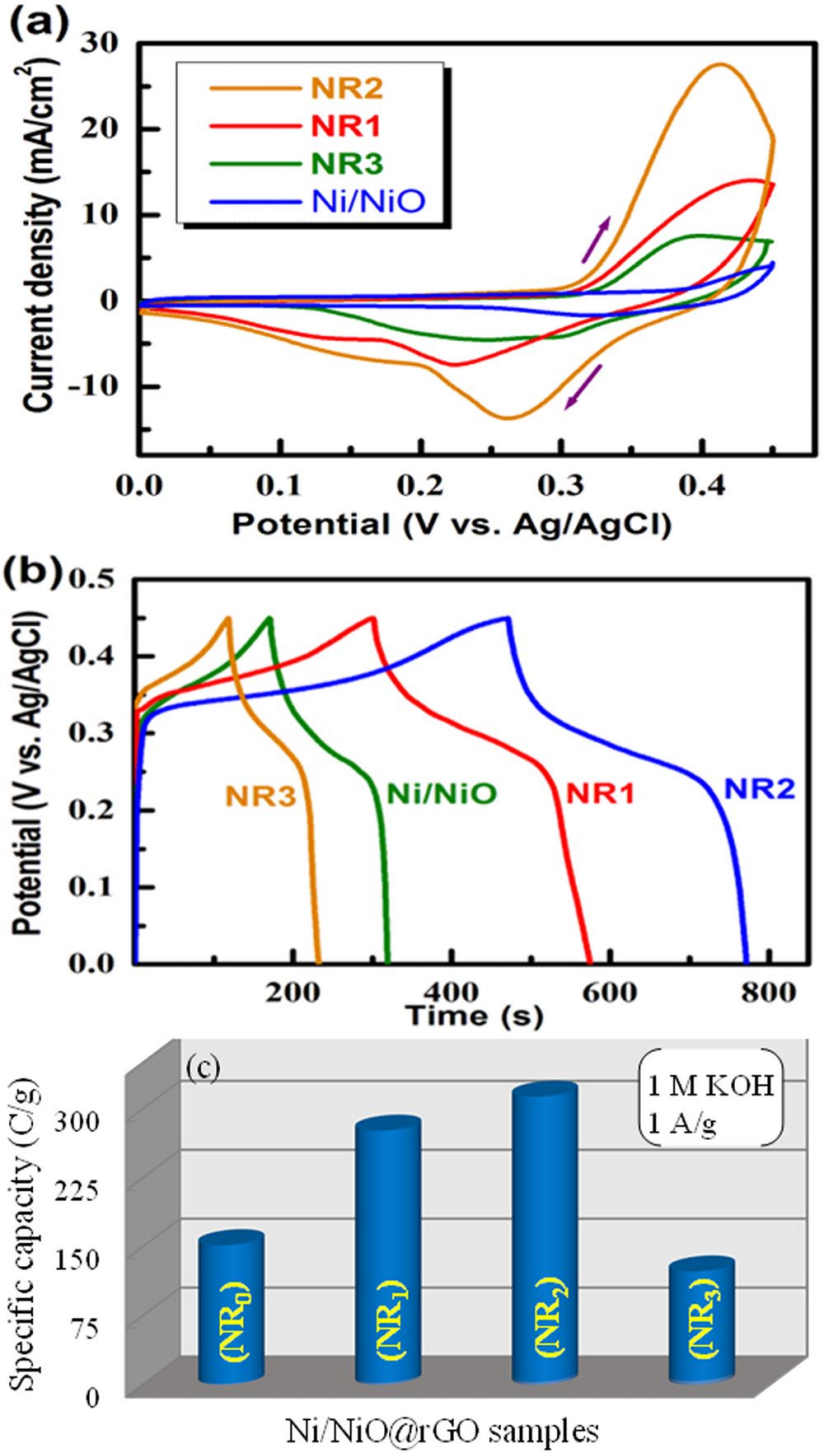
(**a**) Cyclic voltammograms at 5 mV/s, (**b**) Charge-discharge profiles at 1 A/g and (c) Specific capacity recorded for the pristine, NR_1_, NR_2 _and NR_3 _electrodes in 1 M KOH.

To enhance the electrochemical activity of the Ni/NiO sample, each of 5, 15, 25 wt.% rGO was added (the resulting samples were labeled as NR_1_, NR_2_ and NR_3_). Then, the electrochemical activities of the samples were examined in 1 M KOH. Figure 4(a) shows the CV curves recorded for the NR_1_, NR_2_ and NR_3 _electrodes along with the pristine Ni/NiO electrode obtained from the (1:1) mole ratio at a scan rate of 10 mV/s in 1 M KOH. It is seen that all the rGO-added electrodes show clear redox peaks proving the occurrence of Faradaic reaction. On progressive analysis for the three wt.% ratios, it is seen that the NR_2_ electrode shows the highest enhancement of peak current compared to other two samples. This means that the battery-type characteristics can be enhanced by anchoring Ni/NiO grains on to the rGO. Such an enhancement of current is attributed to the presence of the rGO in the sample which can provide a conductive nano-network and more electroactive sites, leading efficient Faradaic reaction. It is noted that even the NR_1_ sample shows better electrochemical activity than that of the pristine Ni/NiO electrode. On the other hand, the NR_3_ electrode shows depletion of peak current. In this case, the large amount of the added rGO (25 wt.%) seems to shield the Ni/NiO grains in such a way that the Ni/NiO grains are not fully involved in the Faradaic reaction as the CV curves are more like EDLC behavior, mainly contributed by the rGO sheets. The CV curves for different scan rates of the NR_1_ and NR_3_ are given in supplementary (Fig. S3).

Figure 4(b) depicts the galvanostatic charge-discharge profiles of the NR_1_, NR_2_, NR_3_ and pristine electrodes recorded at 1 A/g in 1 M KOH. The appearance of a plateau-like profile in the range of 0.32–0.24 V confirms a battery-type behavior in all case. The plateau is well pronounced in the NR_1_ and NR_2_ electrodes confirming the influence of Ni and the rGO on the redox process. It can be seen that the NR_2_ electrode has larger charge/discharge times compared with all other electrodes, substantiating the aforementioned CV data (Fig. 4(a)). This implies that the NR_2_ electrode is capable of accommodating more charge than other electrodes. Thus, the rGO can enhance the battery-type behavior of the base material, (Ni/NiO). Figure 4(c) represents a comparison of the specific capacities for the pristine Ni/NiO (1:1), NR_1_, NR_2_ and NR_3_ electrodes obtained at 1 A/g in 1 M KOH. The observed specific capacities were found to be 150, 275, 310 and 120 C/g for pristine Ni/NiO (1:1), NR_1_, NR_2_ and NR_3_ electrodes, respectively. Undoubtedly, the NR_2_ electrode exhibits nearly double the enhancement of specific capacity. Thus, the specific capacity of the pristine Ni/NiO can be tuned from 150 to 310 C/g by anchoring on to the rGO.

**Figure 5:**
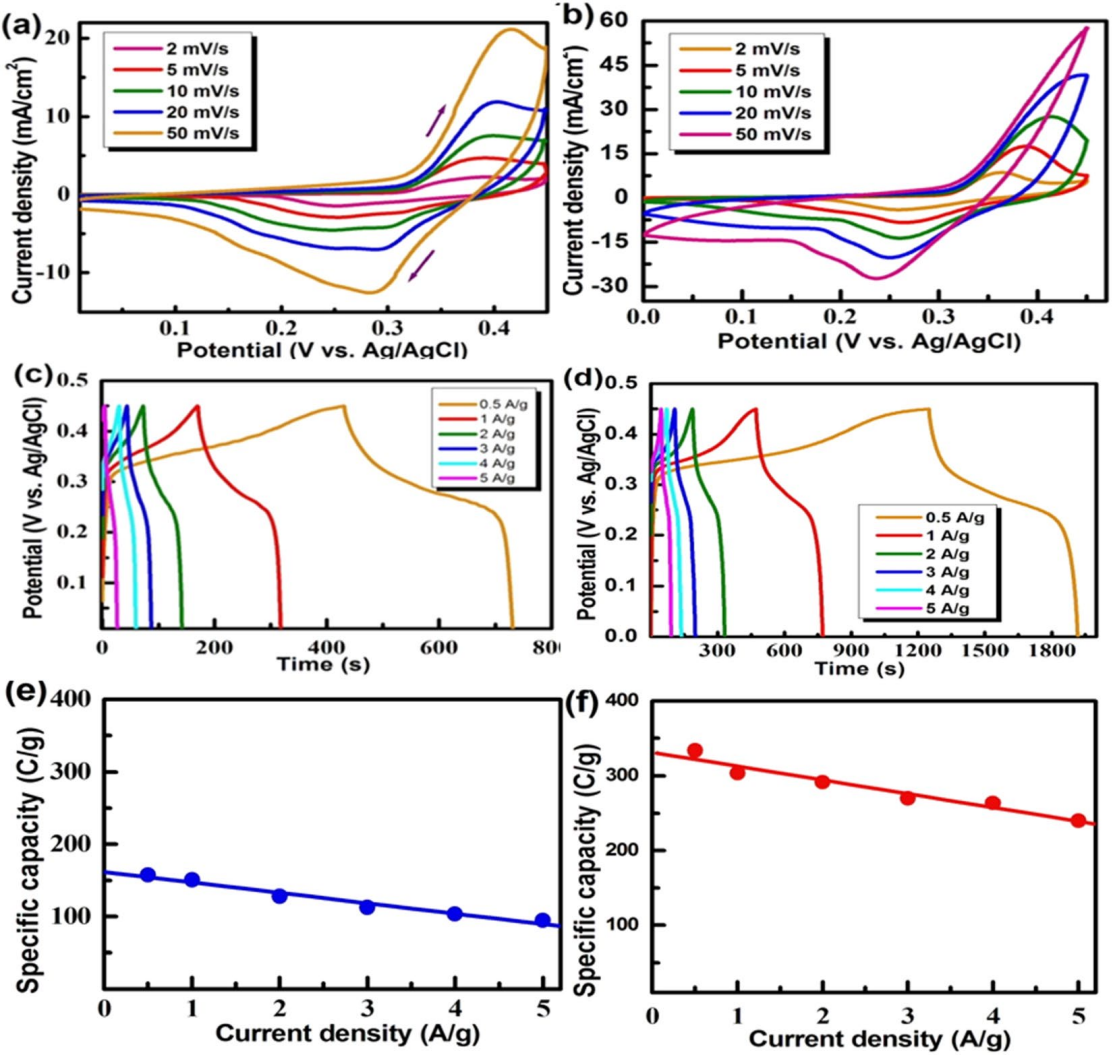
(**a**,**b**) Cyclic voltammograms at various scan rates, (**c**,**d**) Charge-discharge profiles at various current densities and (**e**,**f**) dependence of specific capacityon current density recorded of pristine Ni/NiO(1:1) (left) and NR_2_ (right) electrodes in 1 M KOH.

Figure 5(a,b) show the comparison of CV curves recorded for the pristine Ni/NiO (1:1) and NR_2_ electrodes in 1 M KOH in the potential range 0–0.45 V at various scan rates. For both the electrodes, the oxidation peak is seen at 0.36 V and the reduction peak is seen at 0.27 V with a peak potential difference which resembles a quasi-reversible reaction. It was seen that the peak current (anodic and cathodic) increased with the increase of scan rates which is usually observed. The NR_2_ electrode exhibits large peak currents in all scan rates compared to the pristine Ni/NiO (1:1) electrode. The NR_2_ electrode exhibits more than double the enhancement of peak current at each scan rates. Figure 5(c, d) show the galvanostatic charge-discharge profiles of the pristine Ni/NiO (1:1) and NR_2_ electrodes recorded at different current densities. It is seen that the charge/discharge times of the NR_2_ electrode are much higher than that of the pristine Ni/NiO electrode. Undoubtedly, the NR_2_ electrode exhibits more than double the enhancement of specific capacity at each current density. The combined effects of high conductivity and increased surface area could have led to high electrochemical activity by providing more reactive sites as well as better interaction with the KOH. The conducting rGO sheets would provide a short pathway for easy charge transport in the Ni/NiO matrix, while the high surface area would have led to more reaction sites in the electrode. Figure 5 (e and f) shows a variation of specific capacity (calculated by using Eq. 5) with different current densities recorded for pristine Ni/NiO and NR_2_ electrodes in 1 M KOH. The specific capacity decreases as the discharge current increases which are usually observed for any battery-type material. The declined specific capacity at the high current rate is due to limited usage of the active material. At low current density, all surface of the active material is involved in the charge transporting with the OH^−^ ions, results in an efficient redox reaction and hence high specific capacity. Interestingly, it is noted that the NR_2_ electrode exhibits nearly similar capacity even at high current density; this may be due to the high conductivity of the rGO, enabling efficient charge carrier/transport.

**Figure 6:**
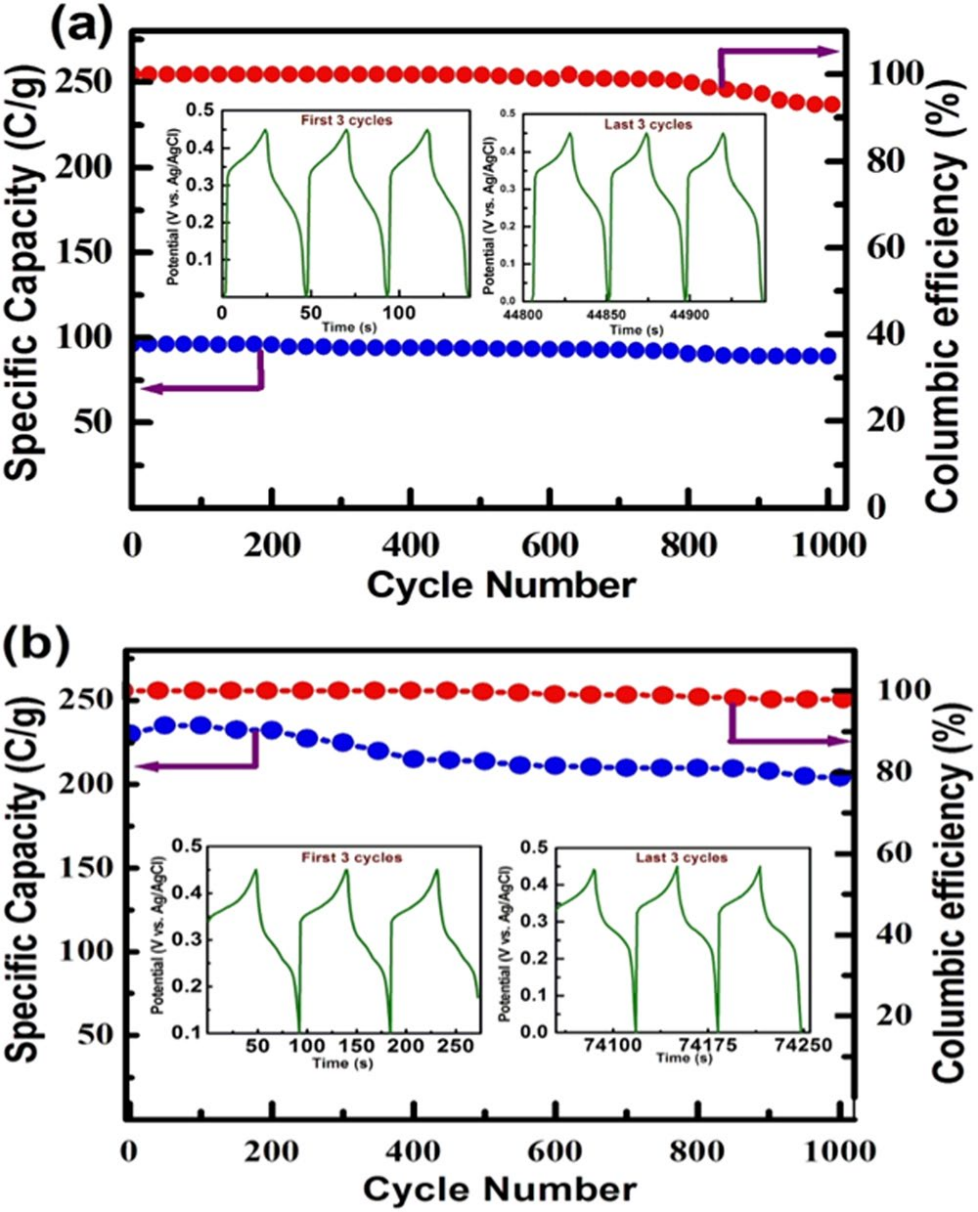
Life-cycle data and Coulombic efficiency recorded at 5 A/g in 1 M KOH for (**a**) pristine Ni/NiO (1:1) and (**b**) NR_2 _electrodes.

The stability of the electrode is one of the important parameters for developing a hybrid-energy storage device. Figure 6 (a and b) show the comparison of life-cycle data along with Coulombic efficiency recorded for pristine Ni/NiO and the NR_2_ electrodes at a current density of 5 A/g in 1 M KOH. The insert in the Fig. 6 (a and b) show the first three and last three cycles of charge-discharge profiles for the respective electrodes. It is seen that the NR_2_ electrode exhibits specific capacities of 225 and 210 C/g at 1^st^ and 1000^th^ cycles, respectively. This implies that the NR_2_ electrode retains as high as 95% capacity at 1000^th^ cycle. On the other hand, the pristine electrode exhibits specific capacities 95 and 80 C/g at the 1^st^ and 1000^th^ cycle, respectively, confirming that the pristine electrode retains only 85% of its capacity. Such a remarkable cycle life exhibited by the NR_2_ electrode is due to combined effects of Ni metal and conductive rGO nanosheets. The Coulombic efficiency of the NR_2_ and pristine electrodes were also estimated for 1000 charge-discharge cycles and the obtained results are shown in Fig. 6. It is noted that the NR_2_ electrode exhibits nearly 100% Coulombic efficiency in all the charge-discharge cycles even at the 1000^th^ cycle, implying excellent stability of the electrode. However, a notable decrease in the Coulombic efficiency has been exhibited by the pristine Ni/NiO electrode just after 500 cycles, implying poor stability. Thus, the addition of the rGO, enhanced stability also.

**Figure 7:**
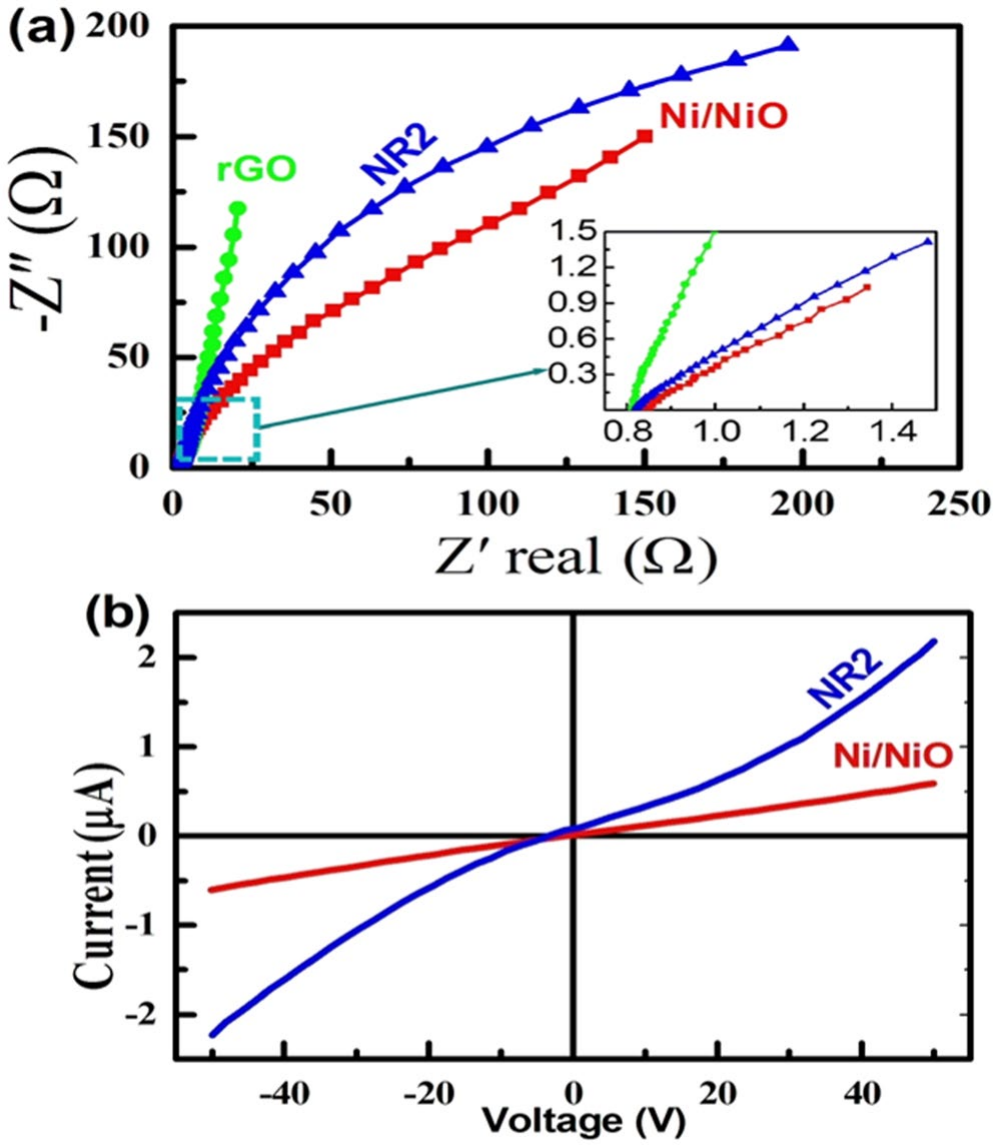
(**a**) Nyquist plots recorded on the rGO, Ni/NiO (1:1) and NR_2_ electrode in 1 M KOH and (**b**) Polarization (I-V) curves ofthe pristine Ni/NiO and NR_2_ samples.

To substantiate the aforementioned increased electrochemical characteristics of the NR_2_ electrode, complex impedance plots were recorded on the NR_2_ electrode in the frequency range of 100 kHz to 10 mHz in 1 M KOH. For comparison, the complex impedance plots of pristine and the rGO electrode were also done. The obtained Nyquist plots are shown in Fig. 7(a). The Nyquist plots consist of three components, solution resistance (R_s_), Charge-transfer (Faradaic) resistance (R_F_) and Warburg component (W). Similar observations have been reported elsehwere^31,32^. It can be seen from the Nyquist plots that the Faradaic resistance is in the order of rGO > NR_2_ > Ni/NiO. Undoubtedly, the resistance of the rGO is very low as expected due to its high conductivity. Thus, the addition of the rGO to the Ni/NiO has resulted in enhancement of the conductivity (lower Faradaic resistance) resulted in better electrochemical activity. The impedance data have been substantiated by the current-voltage (I-V) curves shown in Fig. 7(b). It is seen that the I-V curve of the pristine Ni/NiO sample is linear and less conductive than that of the NR_2_ electrode which is non-linear and more conductive^33^. Thus, the enhanced electrochemical activity of the rGO-added Ni/NiO electrode (NR_2_) is due to the improved conductivity leading to the better Faradaic reaction, confirming that the Ni/NiO@rGO nanocomposite can be a promising electrode for the hybrid energy-storage application.

### EDLC performances of rGO

**Figure 8:**
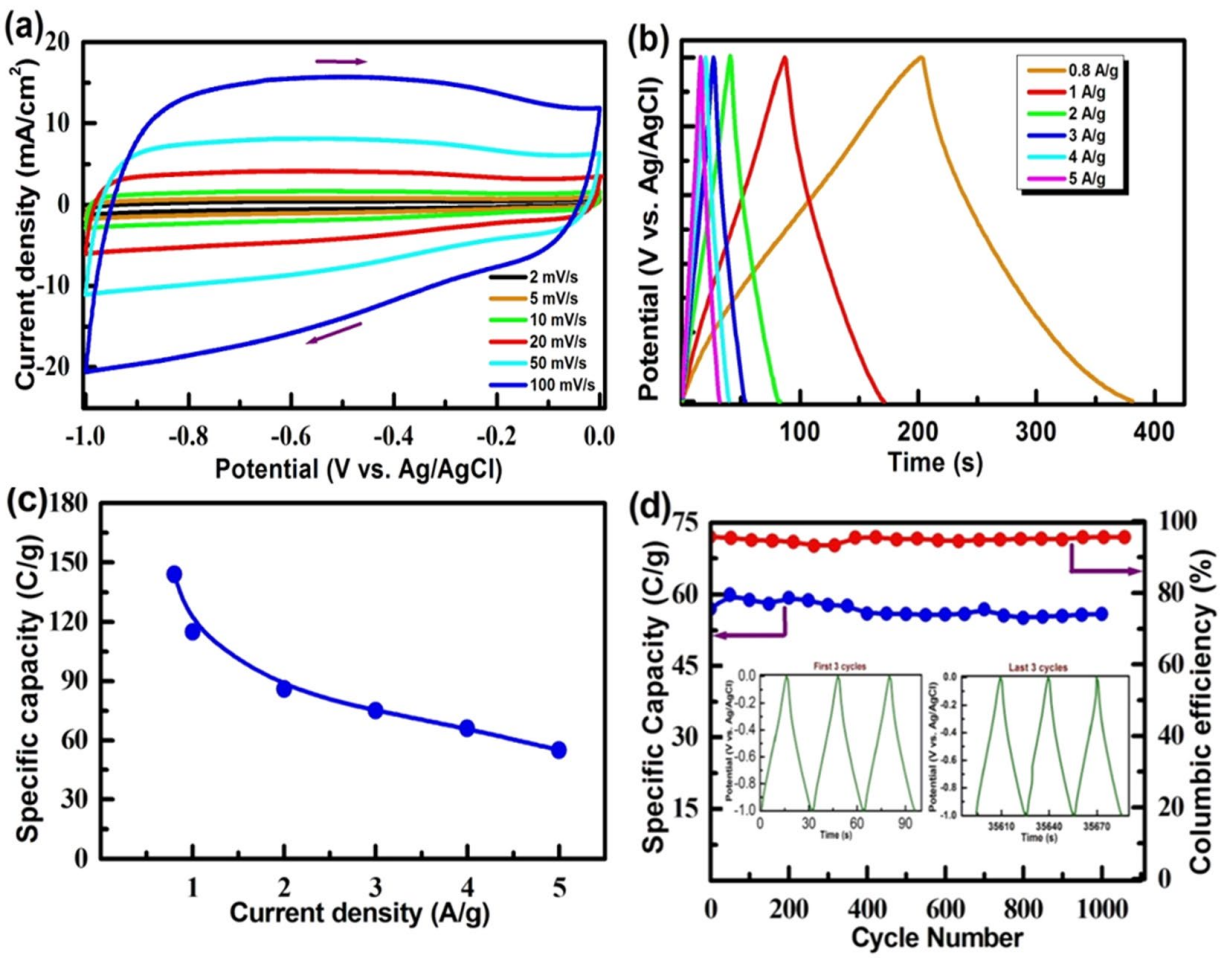
(**a**) Cyclic voltammograms at various scan rates, (**b**) Charge-discharge profiles at different current densities, (**c**) Variation of specific capacitance with current density and (**d**) Life-cycle data as well as Coulombic efficiency recorded for the rGO electrode in 1 M KOH.

The EDLC property of the rGO was initially assessed by CV studies in the potential range of −1 to 0 V at different scan rates in 1 M KOH. The obtained CV curves have been shown in Fig. 8(a). A clear nearly rectangular shape CV curves have been observed in all the scan rates. The appearance of the rectangular shape CVs and absence of redox peaks confirm that the capacitor behavior is mainly contributed by double layer formation (non-Faradaic). Figure 8(b) shows the charge-discharge profiles of the rGO electrode recorded at different current densities in 1 M KOH. The charge-discharge profiles are mirror images in all currents substantiating the CV data. As the current density increased the charge-discharge times decreased significantly. The specific capacitances were obtained from the charge-discharge profiles at different current densities by using the following formula:

5 $$C{}_{sp}\,=\frac{I\times t}{m\times v}\,F/g$$

where *C*_*sp*_ is specific capacitance (F/g), *I* is the current (A), *t* is discharge time (s), *m* is active mass (g), *V* is a potential window (V). The obtained data are displayed in Fig. 8(c). The specific capacitances of the rGO were estimated to be 145, 105, 86, 75, 67, 55 F/g at 0.8, 1, 2, 3, 4, 5 A/g current densities, respectively. At lower current density, high specific capacitance and vice-versa has been observed. Figure 8(d) represents the life-cycle data and Coulombic efficiency of the rGO electrode. The insert shows the first and the last three cycles of charge-discharge profiles. The rGO retained almost similar specific capacity in all cycles. In addition, it was found that the rGO exhibits more than 95% Coulombic efficiency and remains almost constant throughout the examined 1000 cycles. Such a good stability has been shown by a carbon-based electrode which has been ascribed to highly fold curtain-like and non-agglomerated graphene sheets. The specific capacitance in the range of 100–245 F/g has been reported for the rGO prepared by other authors^4,5,27,33^.

### Performances of solid-state hybrid device consisting of Ni/NiO@rGO (positive electrode) with rGO (negative electrode)

**Figure 9:**
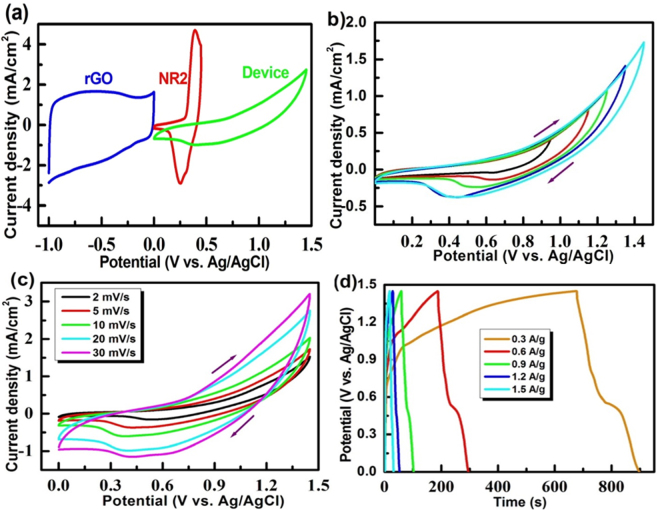
(**a**) CV plotsof the rGO, NR_2_ and solid-state hybrid device, (**b**) CV plots at different potential windows, (**c**) CV plots in the potential range 0–1.45 V at different scan rates, and (**d**) Charge-discharge profiles at different current densitiesrecorded for the device in PVA/KOH gel electrolyte.

A solid-state hybrid energy-storage device was fabricated by using the aforementioned NR_2_ as positive electrode and the rGO as a negative electrode in PVA/KOH gel electrolyte. To ensure equal charge (Q^+^ = Q^−^, where Q^+^ and Q^−^ are the charges of the positive and negative electrodes, respectively), the two electrode masses were balanced. The mass loadings of positive electrode material and negative electrode material were calculated by the following formula^34^:

6 $$\frac{m+}{m-}=\frac{{C}^{-}{V}^{-}}{{C}^{+}{V}^{+}}$$

where *C* ^*−*^and *C* ^+^ represent the specific capacitances (F/g), *V* ^−^ and *V* ^+^ represent the potential windows for the galvanostatic charge-discharge process and *m* ^−^ and *m* ^+^ are the mass loadings of active material on the negative and positive electrodes. Figure 9(a) shows the CV curves for the each of the positive electrode and negative electrode and hybrid device. The CV profile of the device implies that a wide potential window operation is possible for the hybrid device. Figure 9(b) shows CV curves of the hybrid device at different potential windows at a scan rate of 5 mV/s. It can be seen that for the hybrid device, the optimum potential window could be 0–1.45 V. Figure 9(c) shows the CV curves of the hybrid device recorded at different scan rates in the potential range of 0–1.45 V. It is evidently seen that in all scan rates the CV curves have the Faradaic as well as non-Faradaic contributions, confirming perfect asymmetric supercapacitor behavior. As the scan rate increased, the anodic and the cathodic currents increased significantly. Figure 9(d) shows the charge-discharge profiles recorded for the hybrid device in the potential window of 0–1.45 V at different current densities. It is seen that the charge-discharge profiles are in accordance with the CV curves discussed above. There exists clear plateau between 0.6 to 0.4 V, substantiating the CV data (Fig. 9(c)).

**Figure 10:**
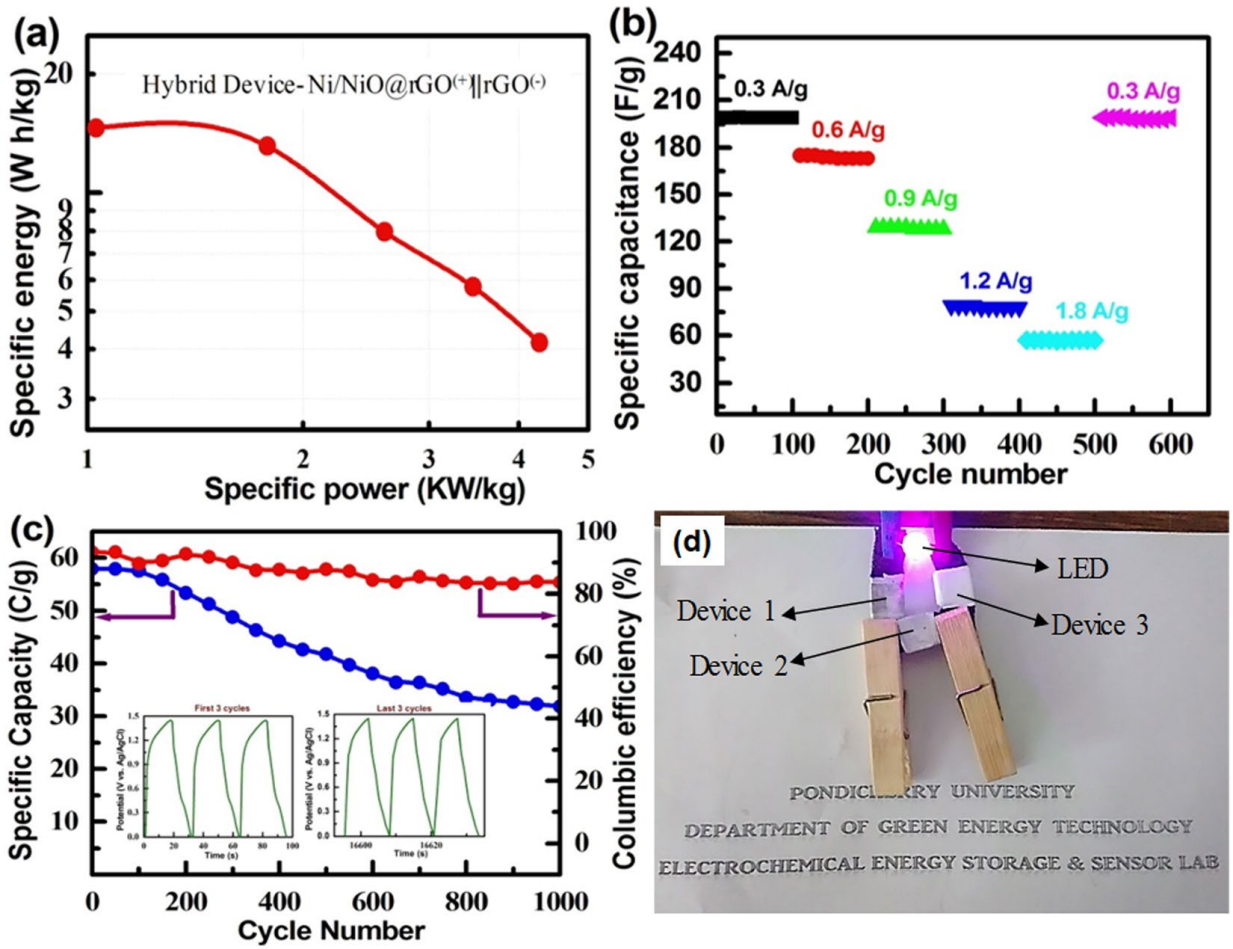
(**a**) Ragone plot, (**b**) stability studies at different current densities, (**c**) cycle-life data and Coulombic efficiency recorded for the hybrid device consisting of the NR_2 _positive electrode and rGO negative electrode in gel-type electrolyte and (**d**) photograph of the fabricated three hybrid devices lighting an LED.

From the charge-discharge profiles, the energy density (*E*) and power density (*P*) of the hybrid device were calculated by using the following formulae^35,36^:

7 $$E=0.5\times 0.28\,C{V}^{2}\,Wh/\text{kg}$$

8 $$\,P=\frac{E\times 3600}{{\rm{\Delta }}t}\,{\rm{W}}/{\rm{kg}}$$

where *E* is specific energy (Wh/kg), *C* is specific capacitance (C = 4C_s_/∆V, C_s_ = specific capacity), *∆V* is potential window and *P* is specific power (W/kg). Figure 10(a) shows the Ragone plot obtained for the hybrid device. It can be seen that the hybrid device exhibits high energy density of 14.6 W h/kg, along with high power density of 4.3 kW/kg with a wide potential window of 0–1.45 V. Even at an energy density of 8 W h/Kg, nearly 3000 W/kg power density could be obtained. An energy density in the range of 1.0–1.4 mW/cm^3^ and power density in the range of 0.4–16.5 mW/cm^3^ have been reported for the pseudocapacitor consisting of Ni@NiO nanowire^19,37^. Recently, hydrothermally-driven NiO/rGO showed energy density of 12.8 W h/kg and the power densities of 2875 W/kg^24^. The symmetric capacitor consisting of each of graphene or asymmetric device of NiO||AC had an energy density in the range of 7–10 W h/kg and the power density in the range of 25–65 W/kg^37,38^. It is noteworthy that the present hybrid solid-state (NR2^(+)^ || rGO^(−)^) device exhibits much higher energy and power densities. The hybrid device was also tested at various current rates for several cycles and the obtained data are shown in Fig. 10(b). It is found that the device retains nearly similar charge storage at each of different current densities. A device exhibits excellent repeatability of charge storage features. Figure 10(c) represents the life-cycle data recorded for the hybrid device at 1.5 A/g. The hybrid device exhibits excellent stability as well as Coulombic efficiency even at 1000 cycles. The device retains as high as 60% of its initial capacitance at the end of the 1000^th^ charge-discharge cycle. Thus, the aforementioned studies show that the NR_2 _electrode having the Ni/NiO anchored onto the rGO could be a potential material for the practical hybrid energy-storage device. Three similar hybrid-energy storage devices were fabricated and connected in series to examine the capability of the resultant device to light an LED bulb. It turned out that the device could light the LED bulb for about 6 min in one charge. Thus, the Ni/NiO@rGO in combination with the rGO could be a potential material for the practical hybrid-energy storage device.

## Discussion

The sol-gel derived Ni/NiO nanocomposite electrodes prepared by variation of (Ni^2+^: CA) mole ratio were found to be electrochemically active. The tunable specific capacity has been observed for the Ni/NiO electrodes with Ni/NiO (1:1) electrode exhibiting high specific capacity of 158 C/g in 1 M KOH. The synthesized rGO in this work exhibited a specific capacitance of about 145 F/g. The rGO-anchored Ni/NiO electrode exhibited enhanced electrochemical activity with the specific capacity of 335 C/g. The addition of the rGO to the Ni/NiO has resulted in enhancement of the conductivity resulted in better electrochemical activity. The solid-state hybrid device consisting of the Ni/NiO@rGO and the rGO electrodes resulted in high-energy density and high-power density with excellent stability due to a combination of Faradaic and non-Faradaic reactions. It was demonstrated that the three hybrid devices connected in series could lit the led bulb for about 6 min. thus, the Ni/NiO@rGO could be a potential material for practical energy storage application.

## Experimental

### Synthesis of Ni/NiO nanocomposites

The Ni/NiO nanocomposites were synthesized as reported recently by us elsewhere^39^. In brief, nickel nitrate (Ni(NO_3_)_2_∙6H_2_ O) and citric acid (C_6_H_8_O_7_) of analytical grade (Fisher Scientific 99%) were used as metal-ion precursor and gelling agent, respectively. To vary (Ni^2+^: CA) mole ratio, in this citrate-based sol-gel process, the nickel nitrate concentration was kept constant at 0.1 M and the concentration of the CA was varied to 0.1, 0.2, 0.4, 0.6 and 0.8 M. A suitable quantity of nickel nitrate was dissolved in the deionized water (DW) and stirred at 80 °C for 1 h on a hotplate. After complete dissolution, the citric acid solution was slowly added to the nitrate solution to form sol. After complete addition, the temperature of the solution was increased to 110 °C for nearly 6 h to form the gel. Then, the formed gel was dried at 130 °C for overnight. Finally, the dried gel was calcined at 500 °C for 2 h in air to obtain the sol-gel product.

### Synthesis of rGO and Ni/NiO@rGO nanocomposites

Initially, GO was prepared by modified Hammers method as described below^27^. 1.0 g of graphite flakes and 1.0 g sodium nitrate were mixed in 40 ml of concentrated sulphuric acid in a 500 ml round-bottom flask in an ice bath and kept on magnetic stirring for 30 min. To the cold solution, 3.0 g of potassium permanganate (KMnO_4_, Fischer Inorganics, and Aromatics) was gradually added with constant stirring for 1 h. Then, 80 ml DW was slowly added and the temperature of the bath was raised to 90 °C. After 30 min of vigorous stirring, the content was diluted further by the addition of 200 ml DW followed by 10 ml of hydrogen peroxide (30%) and further stirred for 1 h. Then, the resulting solution was washed repeatedly and centrifuged. The obtained sediment was re-dispersed in water giving GO. Reduced graphene-oxide was obtained by means of chemical reduction of the GO by using hydrazine hydrate (N_2_H_4_∙H_2_O).The GO suspension (100 ml) was mixed with 100 mL DW water and stirred for 30 min at 85 °C. To the hot solution, 1.0 ml of hydrazine hydrate was added and stirred for another 2 h at 85 °C. The obtained mixture was washed with hot water and ethanol to remove excess hydrazine, and finally dried at 60 °C overnight to obtain solid rGO.

Then, the Ni/NiO@rGO nanocomposites with different amount (weight %) of the rGO, were obtained by dispersing the Ni/NiO sample obtained from (1:1) mole ratio and rGO in ethanol (1 mg/ml).Three different compositions of the Ni/NiO@rGO nanocomposites labelled as NR_1_, NR_2_ and NR_3_were prepared by keeping the Ni/NiO amount constant and varying the rGOin different amount, 5, 15, 25 wt.%. The Ni/NiO and rGO mixture was dispersed in the ethanol and subjected for probe sonication for 2 h. Then, the content was dried at 110 °C for 48 h to obtain solid Ni/NiO@rGO sample.

### Material Characterization and Electrochemical Test

The phase analysis of the obtained Ni/NiO, rGO, Ni/NiO@rGO samples was carried out by powder X-ray diffraction (Bruker D8 Advanced) using Cu K_α_ radiation (λ = 1.5418 Å). Raman spectra were recorded by using laser Raman spectroscopy (Witec Confocal Raman instrument with Ar ion laser 784 nm CRM200) in the frequency range of 100–2000 cm^−1^. The Fourier transform infra-red (FT-IR) spectra were obtained by using FT-IR spectroscopy (Thermo Nicolet 6700) in the frequency range of 400–4000 cm^−1 ^by means of KBr pellet technique. The morphology of the samples was observed by using scanning electron microscope (Hitachi S3400N).The electrochemical studies were conducted on stainless steel (SS) substrate. First, the SS surface was made rough to increase the surface area by dipping in conc. H_2_SO_4_ for 1 h and then washed repeatedly with DW as well as acetone. The working electrode slurry was made by mixing each of Ni/NiO, Ni/NiO@rGO or rGO (80 wt.%), carbon black (10 wt.%) as conductivity enhancer and polyvinylideneflouride (PVDF) as a binder (10 wt.%) using N-Methyl-2-pyrrolidone (NMP) as a solvent. The obtained slurry was mechanically pasted on the SS then dried at 100 °C overnight. The estimated active mass on the SS was about 2 mg/cm^2^ in each case. The exposed area of the SS to the electrolyte was 1 × 1 cm^2^ and the rest of the SS area was masked with the Teflon tape. All the electrochemical tests were performed using electrochemical workstation Bio-logic (SP-150). The electrochemical activity of the samples was examined by using cyclic voltammetry and galvanostatic charge-discharge studies in a three electrode cell in 1 M KOH. The cyclic voltammograms (CVs) were recorded in a potential range of 0–0.45 V vs. Ag/AgCl at different scan rates of 2, 5, 10, 20 and 50 mV/s. The galvanostatic charge-discharge cycles were carried out at current densities of 0.5, 1, 2, 3, 4 and 5 A/g. The cycle-life data were collected at a current density of 5 A/g for 1000 charge-discharge cycles. The electrochemical impedance spectroscopy studies were done in the frequency range of 10^5^ Hz – 10^–2^ Hz.The current-voltage (I-V) curves of the samples were measured using source meter (Keithley: 2410-C) in the potential range 50 to −50 V at a scan rate of 100 mV/min.

### Fabrication of Solid-state Hybrid Device

The solid-state hybrid supercapacitor was constructed by using the Ni/NiO@rGO nanocomposite as positive electrode and the rGO as negative electrode in a gel electrolyte. The gel electrolyte was prepared by mixing 0.5 g of polyvinyl alcohol (PVA, Fisher scientific) with 10 ml of DW. The mixture was subjected to 90 °C under stirring for 30 min till a clear solution was obtained and cooled to room temperature. Then, 10 ml of potassium hydroxide (KOH, Fisher scientific) was added to the mixture in drop wise and continued stirring for 1 h. The obtained slurry was poured in to a petri dish and dried naturally. The paper-like dried gel-electrolyte with thickness nearly 1 mm was cut into 2 × 2 cm^2^ and sandwiched between the positive and the negative electrodes. The CV curves at different potential ranges as well as scan rates, and galvanostatic charge-discharge profiles at different current densities, were done on the hybrid device using the same electrochemical workstation.

### Data Availability

All data generated or analysed during this study are included in this published article (and its Supplementary Information files) and some are available with the corresponding author on request.

## Electronic supplementary material

Supplementary data
