# Supplementary material for: Performance of Solid-state Hybrid Energy-storage Device using Reduced Graphene-oxide Anchored Sol-gel Derived Ni/NiO Nanocomposite
Source: Sci Rep. 2017 Nov 10;7:15342. doi: 10.1038/s41598-017-15444-z (PMC5681587; doi:10.1038/s41598-017-15444-z)
Supplement: Supplementary file 1 — Supplementary data [file 41598_2017_15444_MOESM1_ESM.pdf]

Performance of Solid-state Hybrid Energy-storage Device using Reduced Graphene-oxide Anchored Sol-gel Derived Ni/NiO Nanocomposite

Himadri Tanaya Das,[1] Kamaraj Mahendraprabhu, [1†] Thandavarayan Maiyalagan[2]

& Perumal Elumalai[1]*

[1]Electrochemical Energy and Sensors Lab, Department of Green Energy Technology,

Madanjeet School of Green Energy Technologies, Pondicherry University, Puducherry-605014, India.

[2]SRM Research Institute & Department of Chemistry, SRM University, Kattankulathur, 603203, India.

†Present Address: Department of Bioelectronics and Biosensors, Alagappa University, Karaikudi-630003, Tamilnadu, India

Supplementary

Fig.S1

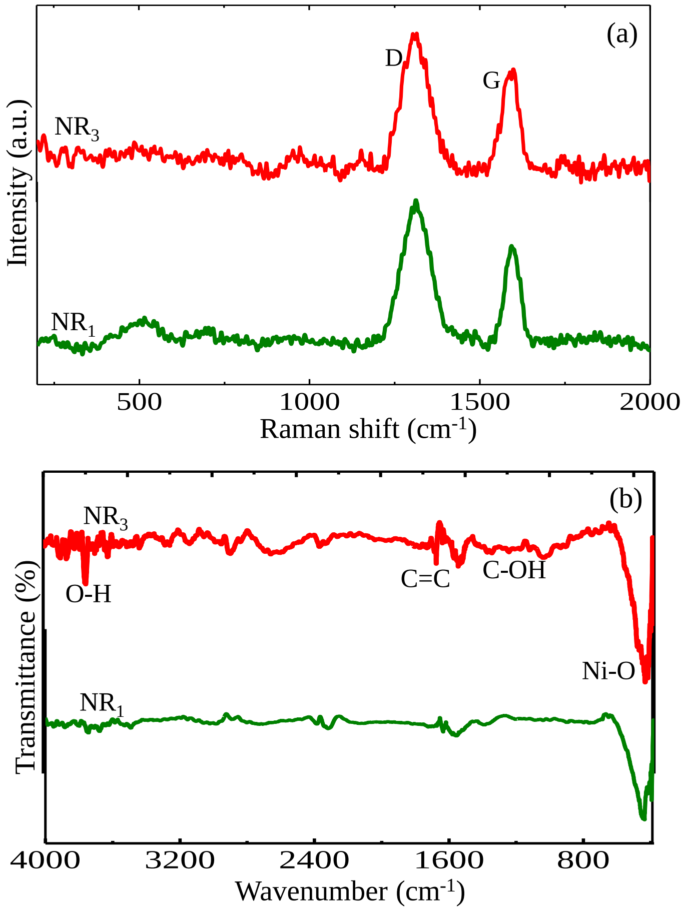

Figure S1. (a) Raman spectra and (b) FT-IR spectra of the NR_1 and NR_3.

The presence of rGO in both NR_1 and NR_3 has been confirmed. A low Raman shift of 500 cm^{-1} obtained for the NR_1 sample confirm that amount of the rGO in NR_1 is less. At high rGO content, the Ni-O shift is not clearly visible, may be due to shielding effect or dilution.

Fig. S2

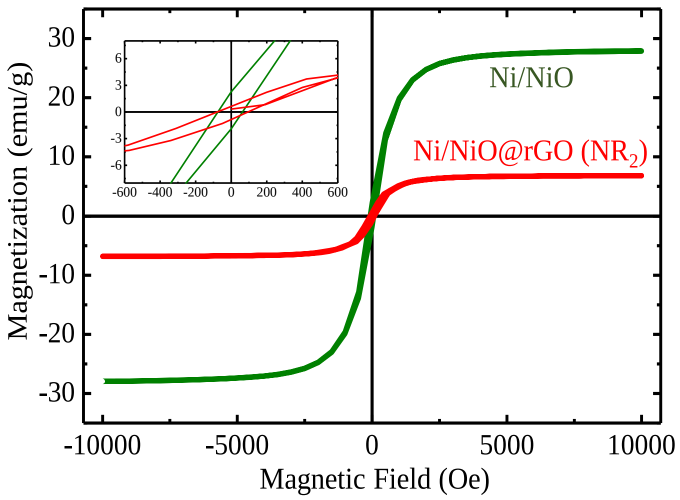

Figure S2 Magnetisation (M-H) curves recorded for curve pristine Ni/NiO and NR_2 sample.

The absence of hysteresis in both cases confirms that the Ni/NiO grains are super-paramagnetic nature due to nano size effect. In both cases, the magnetisation reaches saturation. A large decrease in the magnetisation has been observed if the rGO was added to the Ni/NiO. The rGO sheets may be shielding the Ni/NiO grains resulting in the reduction of saturation magnetization (M_s) from 28 emu/ g to 6 emu/ g. The coercivity of the both the loops (H_c (Ni/NiO) and H_c (NR_2) sample) are found to be 66 and 86 Oe, respectively. While the Remanent magnetization, M_r was found to be 2 emu/ g for the Ni/NiO and 0.4 emu/ g for the NR_2. Thus, the super-paramagnetism seems to be diluted by addition of the rGO, a non-magnetic matrix. There is no magnetic interaction between the rGO and Ni/NiO as the nature of the hysteresis curve did not change, except the magnetic saturation.

Fig. S3

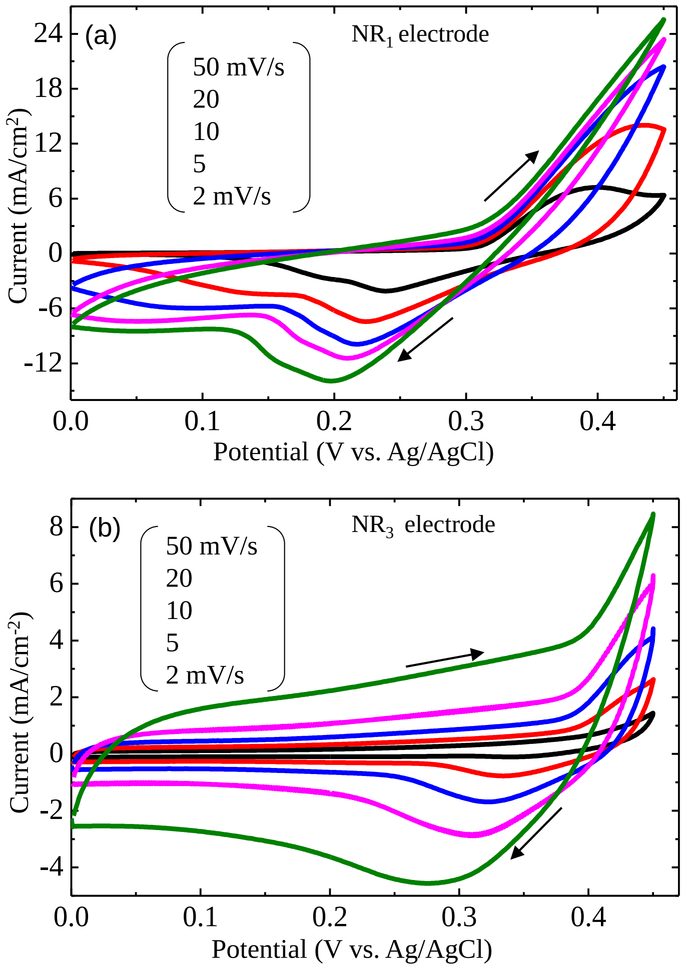

Figure S3. (a) shows the CV curves recorded for NR_1 electrode and (b) gives CV curves for NR_3 electrode at various scan rates in 1 M KOH.

Interestingly, it was found that in higher wt. % of the rGO, the pseudocapacitance nature got submerged which seem to be activated after several charge-discharge cycles, and results in better interaction of Ni/NiO active mass with electrolyte.

Fig. S4

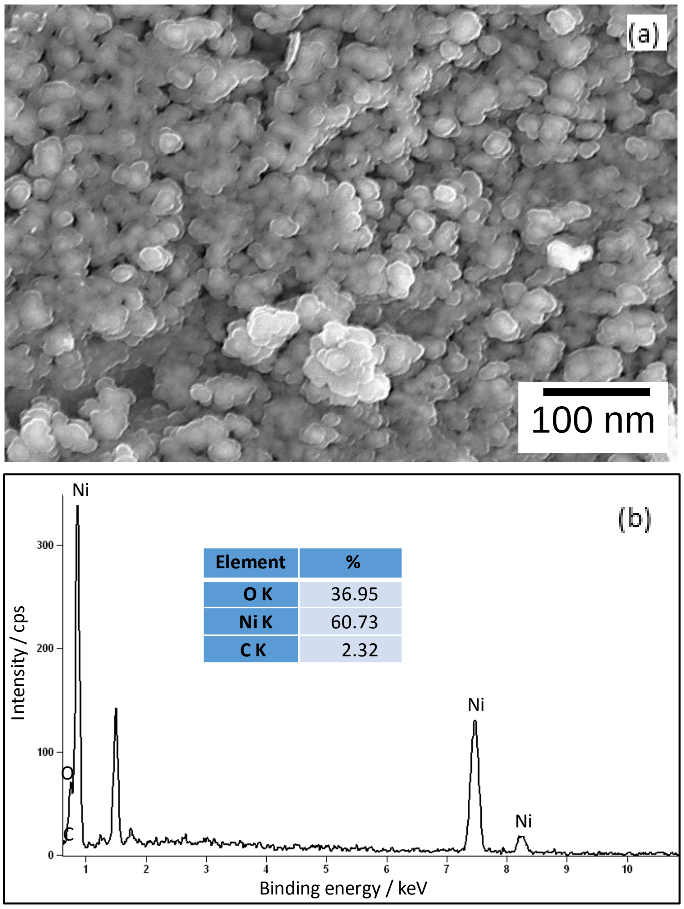

Figure S4. (a) SEM image and (b) EDAX profile recorded on the Ni/NiO sample. Inset: The obtained atomic % of O, C and Ni in the Ni/NiO sample.

The EDAX analysis confirms the presence of predominantly, O and Ni elements in the Ni/NiO sample.
